# Water-stress physiology of *Rhinanthus alectorolophus*, a root-hemiparasitic plant

**DOI:** 10.1371/journal.pone.0200927

**Published:** 2018-08-01

**Authors:** Petra Světlíková, Tomáš Hájek, Jakub Těšitel

**Affiliations:** 1 Faculty of Science, University of South Bohemia, České Budějovice, Czech Republic; 2 Institute of Botany of the Czech Academy of Sciences, Třeboň, Czech Republic; 3 Department of Botany and Zoology, Masaryk University, Brno, Czech Republic; Estacion Experimental del Zaidin, SPAIN

## Abstract

Root-hemiparasitic plants of the genus *Rhinanthus* acquire resources through a water-wasting physiological strategy based on high transpiration rate mediated by the accumulation of osmotically active compounds and constantly open stomata. Interestingly, they were also documented to withstand moderate water stress which agrees with their common occurrence in rather dry habitats. Here, we focused on the water-stress physiology of *Rhinanthus alectorolophus* by examining gas exchange, water relations, stomatal density, and biomass production and its stable isotope composition in adult plants grown on wheat under contrasting (optimal and drought-inducing) water treatments. We also tested the effect of water stress on the survival of *Rhinanthus* seedlings, which were watered either once (after wheat sowing), twice (after wheat sowing and the hemiparasite planting) or continuously (twice and every sixth day after that). Water shortage significantly reduced seedling survival as well as the biomass production and gas exchange of adult hemiparasites. In spite of that drought-stressed and even wilted plants from both treatments still considerably photosynthesized and transpired. Strikingly, low-irrigated plants exhibited significantly elevated photosynthetic rate compared with high-irrigated plants of the same water status. This might relate to biochemical adjustments of these plants enhancing the resource uptake from the host. Moreover, low-irrigated plants did not acclimatize to water stress by lowering their osmotic potential, perhaps due to the capability to tolerate drought without such an adjustment, as their osmotic potential at full turgor was already low. Contrary to results of previous studies, hemiparasites seem to close their stomata in response to severe drought stress and this happens probably passively after turgor is lost in guard cells. The physiological traits of hemiparasites, namely the low osmotic potential associated with their parasitic lifestyle and the ability to withstand drought and recover from the wilting likely enable them to grow in dry habitats. However, the absence of osmotic adjustment of adults and sensitivity of seedlings to severe drought stress demonstrated here may result in a substantial decline of the hemiparasitic species with ongoing climate change.

## Introduction

Plants rely on water for their structure, maintaining a positive pressure (turgor) against their cell walls [1]. Water shortage induces significant stress in plants; stomatal closure and turgor loss are accompanied by suppression of growth and certain physiological processes such as photosynthesis and transport of assimilates [1,2]. Plant water status is usually described by water potential, a measure of water availability in the system (Ψ; [1,2]). One component of Ψ is osmotic potential (Ψ_π_), the water potential of a solution expressing the molar concentration of dissolved substances in the cell. The examination of water relations allows estimating a number of physiological parameters involved in plant adjustment to water stress. In general, plants adjust to water stress by either decreasing Ψ_π_ via accumulation of osmotically active compounds or increasing the elasticity of their cell walls. While the first strategy leads to the increase of turgor and facilitates water uptake from drier soil, the second strategy enables plants to store more water at full turgor, both of them provide plants with the ability to lose more water without losing turgor [1].

Autotrophic plants acquire water directly and exclusively from the surrounding environment but this is not the case of parasitic and hemiparasitic plants. Root hemiparasites acquire virtually all water and mineral nutrients from their hosts through haustorial connection to vascular bundles in host roots [3,4]. In contrast, organic carbon is acquired partly autotrophically from own photosynthetic activity with the host contributing a variable fraction of organic carbon used by the hemiparasite [5,6]. This mixotrophic resource acquisition strategy [7] is highly efficient in *Rhinanthus* species from the Rhinanthoid clade of the *Orobanchaceae* family as it is based on an open direct xylem-to-xylem connection with hosts [8,9]. It was shown to be driven in particular by comparatively high day- and night- transpiration rates in hemiparasites [10–12], lowering thus their water potential to highly negative values and acting as a strong sink [13]. Similarly to all parasitic plants, *Rhinanthus* spp. accumulate osmotics such as sugar alcohols inside cells to maintain low water potential conditions [13,14], which further facilitates the resource flow through haustoria. Moreover, stomata of some hemiparasites are irresponsive to abscisic acid (ABA) and remain permanently open, even under severe water stress [12,15]. Stomatal transpiration and high content of osmotically active compounds are not the only means by which the solute flux is drawn into hemiparasite. Several species from the Rhinanthoid clade actively secrete excess water from hydathode trichomes present on the abaxial leaf sides [16–18] to make the resource acquisition from the host even more efficient.

The genus *Rhinanthus* comprises at least 25 annual species occurring in northern hemisphere [19]. Some of them are most commonly found and studied root-hemiparasites in Europe, colonizing grassland habitats of low to moderate productivity and water availability [19–22]. The performance of *Rhinanthus* spp. was demonstrated to depend on water availability in a non-trivial way. Depending on the ecological context, established *Rhinanthus* plants may be positively or negatively affected by decreased water availability [6,23]. This is rather surprising considering their water-wasting physiological strategy of resource acquisition based on high transpiration. However, experimental evidence [6,23] and occurrence of stable populations of *Rhinanthus* spp. in dry grasslands [22] indicates that they are able to withstand at least moderate water stress. Moreover, wilted *Rhinanthus* fully recovers from severe water stress within several hours after re-watering [24]. This points to the ability to tolerate water stress, even though their stomata do not close under increased ABA concentration. Surprisingly, studies evaluating the water-stress physiology of root hemiparasites are missing.

Here, we examined water-stress physiology of flowering *R*. *alectorolophus* (Scop.) Pollich using a manipulative experiment with high (200 mL) and low (100 mL) irrigation levels, monitoring gas exchange, water relations, stomatal density, and biomass production and its stable isotope composition. We also focused on seedling survival under water stress conditions. The seedlings were watered either once (after wheat sowing), twice (after wheat sowing and the hemiparasite planting) or continuously (twice and every sixth day after that). We hypothesized that i) the survival of hemiparasite seedlings is negatively affected by drought stress, ii) wilted plants of flowering hemiparasites still considerably photosynthesize and transpire, iii) low-irrigated plants osmotically adjust to long-term water deficiency and therefore iv) their photosynthesis and transpiration are suppressed at more negative Ψ_π_ than in high-irrigated plants, v) stable C and O isotopes and stomatal density differ between treatments reflecting the acclimation of water-related physiological processes to prolonged water stress.

## Materials and methods

### Plant material

*Rhinanthus alectorolophus* is an annual hemiparasitic plant of the family Orobanchaceae [25,26]. It grows in open habitats such as meadows and road verges where it parasitizes wide range of host species. *R*. *alectorolophus* reaches an average height of 30 cm and flowers from May to July [27–29]. It used to be considered as an agricultural pest in Central Europe infecting cereal crops [28] and can be easily grown on wheat or maize.

*R*. *alectorolophus* seeds were collected from a natural population near Nenkovice, Czech Republic (49°0’19.8”N, 16°59’54.1”E) from private land as a part of more extensive research conducted at the site. We obtained permission to collect seeds from both the owner and leaseholder of the land where the collection was done. Ripe capsules of *R*. *alectorolophus* were collected by hand in paper bags. The collected material was left to dry in laboratory under ambient temperature for two weeks to release seeds from the capsules. Seeds were further stored at ambient temperature and air humidity until the start of the experimental work. Seeds of wheat (*Triticum aestivum*), which was used as a host species, were obtained from the Krásná Hora nad Vltavou collective farm, Haklovy Dvory, Czech Republic.

### Growth chamber experiments

The experiments were conducted in a growth chamber from January to March 2016. Pre-germinated seeds of wheat were sown in 130 0.8L-pots filled with a mixture of sand and peat (1:1, v:v ratio). All pots contained 0.5 g Osmocote Exact Standard 5–6M fertilizer per liter of substrate and were well watered (200 mL of water/pot). The diurnal light cycle was set to 12 h light/12 h dark. Temperature ranged from 15–17 (dark) to 17–20°C (light). Metal halide lamps provided photosynthetically active radiation (PAR) flux of 200–600 μmol m^–2^ s^–1^ (depending on spatial position). Young seedlings (3 per pot) of the hemiparasite pregerminated on Petri dishes kept at 4°C for three months were transplanted to 110 pots two days after wheat planting. Of these, only a single plant was kept for the experiment while excessive seedlings were removed after a week. The pots were randomly relocated within the chamber table once a week to filter out possible heterogeneity in non-treatment cultivation conditions (mainly PAR flux).

Three contrasting water treatments were established in 30 parasitized pots to study the effect of drought on the survival of *Rhinanthus* seedlings. Ten pots (hereafter referred to as A-pots) were watered only once (after wheat sowing), ten pots (hereafter referred to as B-pots) were watered twice (after wheat sowing and the hemiparasite planting), and ten pots (hereafter referred to as C-pots) were watered as B-pots and every sixth day after that.

The rest of pots (n = 100) were used to study the physiological response of *Rhinanthus* adults to long-term water stress. Twenty pots served as a non-parasitized control. Two water regimes were established after *Rhinanthus* attachment to the host (indicated by rapid leaf expansion of *Rhinanthus*; [30]). High irrigation pots (W+) and low irrigation pots (W–) received 200 and 100 mL of water every fifth to seventh day, respectively. The intervals between irrigation events were determined on the basis of visibly dry soil in W–pots and clear marks of wilting of respective plants. Both W+ and W–pots were irrigated for entire course of the experiment by isotopically constant source water to minimize its effect on the proportion of oxygen isotopes in plant final biomass [31].

### Seedling survival and soil moisture measurements

Survival of *R*. *alectorolophus* seedlings in A, B, and C-pots was daily documented for 17 consecutive days. Dry or heavily-wilted seedlings were assumed to be dead. In addition to that, we measured the relative water content (RWC) of soil in the pots using an HH2 Moisture Meter with an SM200 sensor (Delta-T Devices Ltd, Cambridge, UK). Three measurements per pot were taken every day and their averages are presented. Only pots with seedlings which were considered as alive on the previous day were measured.

### Physiological measurements

Photosynthetic and transpiration rates were measured 46–63 d after *Rhinanthus* transplant at the irradiance of 500 μmol m^–2^ s^–1^ on intact leaves of flowering hemiparasites (13× W+ and 12× W–) with a Li-6400 Portable Photosynthetic System (LI-COR, Lincoln, USA). The measurements were conducted between 0900 and 1930h on partially dehydrated plants (at least 2 d after the last watering), hereafter referred to as drought-stressed plants. Some of these plants were wilted indicating that they have already undergone turgor pressure loss. Chamber CO_2_ concentration and block temperature were set to 400 μmol mol^–1^ and 20°C, respectively. The relative air humidity inside the Li-6400 chamber was controlled at 60–75%. After finishing the measurements, all measured plants were watered and covered with a plastic bag until additional gas-exchange measurements of fully water-saturated plants, hereafter referred to as water-saturated plants, on the following day. These measurements were done in a same way as previous ones and one leaf per plant was sampled for osmotic potential (Ψ_π gas-exchange_) determination after finishing the measurements. Two plants (1× W+ and 1× W–) did not recover from water stress experienced during the first measurements and were therefore excluded from the data set.

The actual Ψ_π gas-exchange_ of sampled plant parts was measured using thermocouple psychrometry [32]. Leaf samples were cut, immediately sealed in a 2-mL syringe, and frozen at –20°C. The samples were allowed to thaw for maximum 60 min before the start of the measurements. The freeze–thaw cycle disrupted the cell membranes and allowed squeezing the cytoplasm. About 7 μL of the fluid was pipetted onto a cellulose filter paper disc, placed in a 1.25 mm deep sample holder, and enclosed inside the C-52 sample chamber linked to a Wescor HR-33T microvoltmeter (Wescor Electronics, Logan, UT, USA). The air Ψ in the sample chamber equilibrated within 5 min. Measurements were calibrated using 0.3 M NaCl (Ψ_π_ = –1.37 MPa).

The second part of parasitized pots (10× W+ and 9× W–) was subjected 48–62 d after *Rhinanthus* transplant to water potential (Ψ) measurements using a pressure chamber (The Plant Water Status Console, Model 3000; Soil Moisture Equipment Corp., Santa Barbara, USA). The pressure-chamber method measures the decline in leaf Ψ with ongoing leaf dehydration [32–34] enabling construction of the pressure–volume (p–v) curves and Höfler diagrams (Figure A in S1 Fig) providing detailed information about the water relations of measured plants. Upper part (up to 20 cm) of fully water-saturated plants were gently blotted up with cotton sheets to remove droplets of external water, cut, immediately wrapped in stretch film to prevent water loss via transpiration during measurements, weighed, and sealed into the pressure chamber. The pressure–volume data (as described in detail below) were collected using a “squeeze method” to prevent damage to the soft herbal tissue. Briefly, the water loss was induced by pressurization of the chamber with synthetic air and the sap squeezed at each balance pressure (steps of about 0.2 MPa) was collected and weighed, while the plant remained enclosed in the chamber [33].

### Evaluation of pressure–volume curves

We plotted a p–v curve for each measured plant. The p–v curves show the relationship between the inverse of the balance pressure and the cumulative volume of cell sap squeezed, which was then replaced by RWC. Using the “squeeze method” instead of repeated pressurizing was the only way how to avoid mechanical damage to the soft herbal tissue; however, the method generated some identifiable artefacts that required further data processing. The values of RWC after turgor loss were slightly overestimated in most samples because not all the water had been squeezed from the plant before switching to higher balance pressure. This overestimation resulted in steeper slope of the linear part of p–v curves (representing Ψ_π_) and thus an overestimated intercept with x-axis (RWC) and underestimated Ψ_π_ at full turgor (Ψ_π FT_; at RWC = 1). The intercept with x-axis denotes the volume of apoplastic water (RWC_AW_), which usually represents 3–50% of the total volume of water in a leaf [32]. Our values ranged between unrealistically high 35 and 74%, representing unlikely high variability in single species. To reduce these artefacts, we fixed the intercept of all curves at RWC of 23.1% (Figure B in S1 Fig). This value corresponded to such slopes of the linear parts of all the p–v curves that yield mean Ψ_π FT_ of –1.38 MPa (*y*-intercept in Figure B in S1 Fig), which is the mean value measured by thermocouple psychrometry in water-saturated leaf samples collected before pressure-chamber measurements (thermocouple psychrometry measurements were thus used to calibrate the pressure-chamber measurements). Solver module of MS Excel was used to find the slopes. Moreover, as the turgor ceased very slowly (hyperbolically), it was difficult to determine the turgor loss point accurately (Figure B in S1 Fig). In order to reduce the variability due to this inaccuracy, we defined a corrected turgor loss point (TLP_cor_) so that ψ_π_ at TLP_cor_ (ψ_π TLPcor_) and RWC at TLP_cor_ (RWC_TLPcor_) corresponded to the values at 10% of full turgor (Figure A in S1 Fig). The modulus of elasticity (ε) was defined as a slope of the turgor–RWC relationship (between full turgor and the point preceding TLP_cor_).

### Stable isotope analyses

Above-ground biomass of flowering hemiparasites and parasitized wheat (n = 44, 23× W+, 21× W–) were harvested after finishing the measurements, i.e. 48–63 d after *Rhinanthus* planting. Above-ground biomass of control wheat (n = 20, 10× W+, 10× W–) were harvested 62 d after the planting. Biomass samples were dried at 80°C for 48 hours and weighed. Newly-grown leaves of both species were sampled to separate paper bags from 20 parasitized pots and all controls, dried, ball-milled, and embedded in tin capsules for stable isotope analysis of carbon.

Stable isotopes of oxygen were analyzed from α-cellulose isolated from the subset of these samples. The isolation of α-cellulose started by placing milled leaves (30–50 mg) in 15-mL plastic centrifuge tubes and washing them in 8 mL of 80% acetone for 15 min. The tubes were then centrifuged (12 min at 4000 ×*g*), the pellet was resuspended in 8 mL of distilled water, and the tubes were placed to water bath at 75°C. After addition of 80 μL of glacial acetic acid and 160 μL of 25% sodium chlorite, the tubes were incubated for 1 h and the addition of sodium chlorite was repeated. After 2 h, the addition of acetic acid and two subsequent additions of sodium chlorite were repeated and the tubes were incubated once more for 1+2 h. The tubes were vortexed every 30 min during the total 6 h of extraction. Cooled tubes were repeatedly centrifuged and washed in distilled water to obtain clean holocellulose pellet, which was subsequently resuspended in 8 mL of 4.2 M KOH and kept at 22°C for 2 h. Finally, the tubes were centrifuged and the pellet of α-cellulose was successively washed with 2% HCl, water, and acetone, dried at 50°C, and weighted to silver capsules.

The stable isotope analyses were conducted with a PDZ Europa ANCA-GSL elemental analyzer interfaced to a PDZ Europa 20–20 isotope ratio mass spectrometer (Sercon Ltd., Cheshire, UK) at the Stable Isotope Facility at University of California, Davis, USA. Isotopic compositions of the biomass samples were expressed as the δ values reflecting the isotopic difference between the sample and relevant international standards, V-PDB (Vienna PeeDee Belemnite) for carbon and SMOW (Standard Mean Ocean Water) for oxygen.

### Stomatal density

Leaves (n = 14, 9× W+, 5× W–) and bracts (n = 16, 9× W+, 7× W–) of the hemiparasite were examined for stomatal density. Stomatal impressions of the adaxial and abaxial leaf and bract sides were taken by transparent nail polish and observed on a slide by an Olympus CX41 Microscope (Olympus Imaging America Inc., Center Valley, Pennsylvania, USA) and INFINITY1-3C 3.1 MP CMOS Color Camera (Lumenera Corp., Ottawa, Canada). Stomata were counted at 200× magnification from 5 microscopic fields per leaf/bract side of a plant. The number of stomata on the area of 0.325 mm^2^ corresponding to an examined microscopic field was converted to the number of stomata per mm^2^. It should be noted, that stomatal density was difficult to analyze, in particular due to the presence of hydathode trichomes on abaxial bract and leaf sides.

### Statistical analysis

Seedling survival was analyzed by estimating the survival curves by Kaplan-Meier survival function [35] for each water treatment. Comparison between the treatments was performed by a Mantel–Haenszel test [36]. Biomass and isotope data were analyzed using linear models. The biomass data of *Rhinanthus* adults were fitted by a linear model with day after transplant as a predictor to estimate their biomass 60 d after *Rhinanthus* transplant (i.e. when the control pots were harvested). This estimate was used as a response in further statistical modeling of the hemiparasite biomass to minimize the effect of different harvest dates. We did not apply this correction to parasitized wheat due to low correlation of its biomass with harvest day, presumably caused by differential growth dynamics in individual treatments. Biomass data were logarithmically transformed before analysis. We used linear models to test the effects of irrigation treatment, infection by the hemiparasite and their interaction on above-ground-biomass production and stable-isotopic composition of the wheat host. Linear models were also used to test the effect of irrigation treatment on the same biomass parameters of the hemiparasite. Gas-exchange parameters were tested by linear mixed-effect models containing irrigation treatment, osmotic potential, and their interaction as fixed-effect predictors and plant identity as a random factor. Stomatal densities of the hemiparasite were tested separately for adaxial and abaxial sides by linear mixed-effect models with irrigation treatment, bract vs. leaf sample, and their interaction as a fixed-effect predictors and plant identity as a random factor. The differences in water-relation parameters were analyzed by two-tailed t-tests. All statistical analyses were conducted and visualized in R software [37], R packages *survival* and *nlme* were used for survival analysis and linear mixed-effect models.

## Results

### Seedling survival

**Fig 1:**
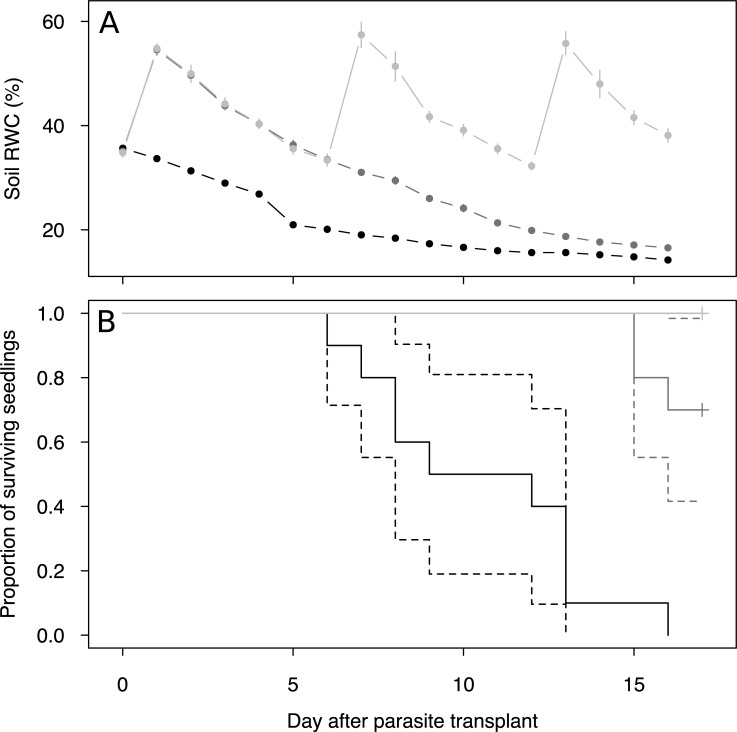
**Soil relative water content (A, soil RWC) and survival of hemiparasitic *Rhinanthus alectorolophus* 0–17 days after its transplant (B) to the pots with the host, *Triticum aestivum***. Black lines represent pots watered only once (after host planting, A-pots), dark grey represents pots watered twice (after host and parasite planting, B-pots), and light grey represents pots watered regularly. Day averages of four soil RWC measurements per pot ± 1.96 standard error are displayed (A). Dashed lines represent 95% confidence intervals (B). n = 10 for each water treatment in the beginning of the experiment.

Survival of parasite seedlings differed among pots (*χ*^2^ = 34.9, df = 2, P<0.001). The seedlings from A-pots started to die 8 d after single watering event (6 d after parasite transplant) at average soil RWC of 20.1% and they were not able to survive more than 18 d after single watering event (2 d before the day 0; Fig 1). Compared with A-pots, B-pot seedlings started to die 15 d after the second watering event (indicated by an increase in soil RWC from 0 d to 1 d; Fig 1) at 17.1% of soil RWC. Second watering event delayed the onset of survival decline by 9 d. All seedlings from non-stressed C-pots survived 17 d after their transplant, when the experiment was terminated (Fig 1B). Host plants showed no distinctive signs of water deficiency and all of them survived till the harvesting times.

### Biomass

**Fig 2:**
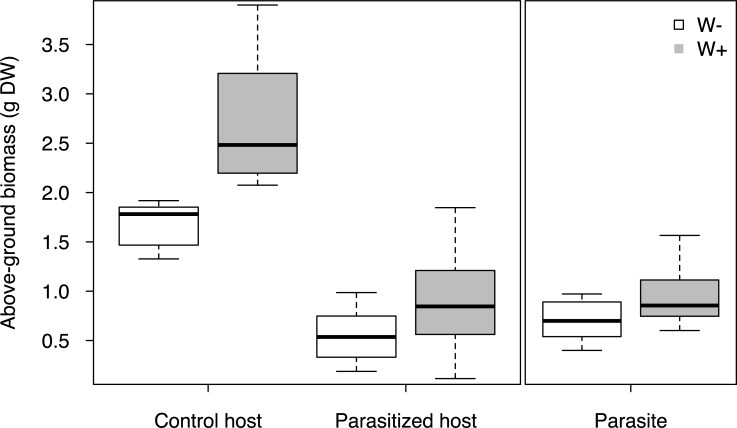
Above-ground biomass of control and parasitized host, *Triticum aestivum*, and the hemiparasite, *Rhinanthus alectorolophus*, grown under high (W+) and low irrigation treatments (W–). n = 10 for W+ and W–unparazitized control pots, n = 21 (W+) and n = 23 (W–) for parasitized pots.

Above-ground biomass production of flowering parasites, as well as control and parasitized host from W–pots was significantly lowered by long-term water stress (Table 1; Fig 2; S1 and S2 Tables). Harvest day had no significant effect on dry mass weight of the host (S1 Table). Parasitism and irrigation treatment markedly affected host biomass (Table 1; S1 Table). Parasitized hosts visibly suffered from water shortage, especially under W–treatment, causing many of their leaves to dry (Figures A-D in S2 Fig).

**Table 1:** Summary of linear models describing the effects of irrigation treatment and infection by the hemiparasite on host *Triticum aestivum* and hemiparasite *Rhinanthus alectorolophus* above-ground biomass production and their stable-isotopic composition.

	Host				Hemiparasite		
*Effect*	Biomass (parasitized and control)	Control biomass	δ^13^C	δ^18^O	Biomass	δ^13^C	δ^18^O
Irrigation treatment	** W+	*** W+	*** W–	*** W–	*** W+	** W–	^+^ W–
Infected	*** ↓		*** ↑	n.s.			
Treatment × Infected	n.s.		n.s.	n.s.			

*** P ≤ 0.001

** P ≤ 0.01

^+^ P = 0.051.

Arrows indicate positive (up) and negative (down) relationship between the response variable and related effect. W+/W–indicate the irrigation treatment with higher values of response variables. Factor Infected represents the effect of parasitic infection on host parameters. δ^13^C and δ^18^O represent the isotopic composition of host overall biomass and hemiparasite biomass. n.s. indicates non-significant terms omitted from the final models. The effects not tested for a particular variable are indicated by light grey. More information in full anova tables (S1 and S2 Tables).

### Physiological measurements

**Fig 3:**
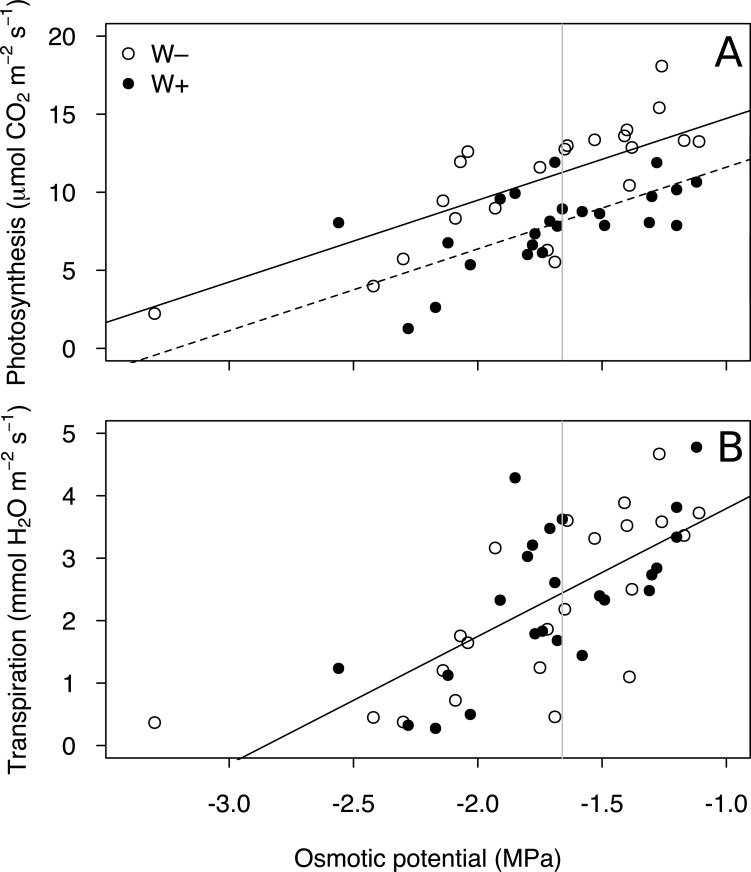
**Photosynthetic (A) and transpiration rates (B) at the irradiance of 500 μmol m**^**–2**^ **s**^**–1**^ **in the leaves of flowering *Rhinanthus alectorolophus* as a function of osmotic potential.** Plants were grown under high (W+) and low irrigation treatments (W–). Points correspond to individual plants, n = 11 and n = 10 for W+ and W–. Each plant was measured twice: when drought-stressed and water-saturated. Linear regression lines are presented by solid and dashed lines (df = 5; LR = 42.15, P<0.0001 (A); df = 4, LR = 31.00, P<0.0001 (B)). Grey vertical line relates to average osmotic potential at turgor loss point (Ψ_π TLPcor_ = –1.66 MPa; Figures A and B in S1 Fig; Table 1) determined by pressure-chamber measurements.

Rates of photosynthesis and transpiration were positively correlated with leaf osmotic potential (Ψ_π gas-exchange_; t_22_ = 7.18; P<0.001; t_22_ = 6.51; P<0.001, respectively; Fig 3; S3 Table). Regardless the irrigation treatment, both processes were significantly lowered in drought-stressed plants (linear models; t_22_ = –5.60, P<0.001 and t_22_ = –7.38, P<0.001 for photosynthesis and transpiration, respectively) compared with water-saturated plants (S4 Tab), which had greater Ψ_π gas-exchange_ (Table 2). Drought-stressed plants displayed low ψ_π_, but still exhibited substantial rates of photosynthesis and transpiration (Table 2). Moreover, photosynthetic rate was higher in the plants grown under W–treatment compared with those of similar ψ_π_ from the W+ treatment (Table 2; Fig 3; S3 Table). We did not find such relationship for transpiration rate (Table 2; Fig 3; S3 Table). Measured plants from contrasting irrigation treatments did not significantly differ in their average Ψ_π gas-exchange_ (–1.70 (W+) and –1.76 (W–)).

**Table 2:** Physiological traits (means ± SE) of hemiparasitic *Rhinanthus alectorolophus* grown under two contrasting irrigation treatments, high (W+) and low (W−).

		Gas-exchange and osmotic potential			Pressure-chamber parameters			
Irrigation treatment	Plant water status	Photosynthesis (μmol CO_2_ m^-2^ s^-1^)	Transpiration (mmol H_2_O m^-2^ s^-1^)	Ψ_π gas-exchange_ (MPa)	Ψ_π FT_ (MPa)	Ψ_π TLPcor_ (MPa)	RWC_TLPcor_ (%)	ε
W+	Drought-stressed	6.8 ± 0.9	1.88 ± 0.36	–1.98 ± 0.08	–1.39 ± 0.03	–1.66 ± 0.03	88.9 ± 0.7	12.5 ± 1.1
	Water-saturated	9.1 ± 0.4	2.91 ± 0.27	–1.42 ± 0.06				
W–	Drought-stressed	8.6 ± 1.1	1.05 ± 0.19	–2.08 ± 0.15	–1.37 ± 0.04	–1.66 ± 0.03	87.4 ± 1.0	11.6 ± 1.4
	Water-saturated	12.9 ± 0.9	3.38 ± 0.22	–1.44 ± 0.08				

Gas exchange was measured at the irradiance of 500 μmol m^–2^ s^–1^ in plants under water stress (drought-stressed) and in the same, but fully water-saturated plants (water-saturated). Leaves subjected to gas-exchange were immediately sampled for actual osmotic potential (Ψ_π gas-exchange_). Pressure-chamber parameters were calculated from pressure-chamber measurements initiated on fully water-saturated plants. We used a corrected turgor loss point equalling to 10% of full turgor (Ψ_π TLPcor_), as the RWC decrease in p-v curves (Figure B in S1 Fig) was hyperbolic and the turgor loss point was hard to determine. Ψ_π FT_ = osmotic potential at full turgor (RWC = 100%), Ψ_π TLPcor_ = osmotic potential at corrected turgor loss point, RWC_TLPcor_ = relative water content at corrected turgor loss point, ε = modulus of elasticity. n = 13 and 12 for gas-exchange and osmotic potential measurements of W+ and W–plants; n = 10 and 9 for pressure-chamber measurements of W+ and W–plants.

Pressure-chamber measurements showed no apparent difference between W+ and W–plants in their water-relation parameters (Table 2; Figures A and B in S1 Fig), including ψ_π FT_, ψ_π TLPcor_, RWC_TLPcor_, and ε. These measurements enabled us to determine actual water status of the plants, in which gas-exchange was measured, by projecting ψ_π TLPcor_ into Fig 3. It is clear from the figure that even wilted plants (Ψ_π gas-exchange_ < ψ_π TLPcor_) were still able to carry out photosynthesis and transpiration.

Despite the absence of osmotic adjustments, wilted hemiparasites recovered very fast from severe drought stress after re-watering (Figures B and D in S2 Fig). Photosynthetic and transpiration rates of W+ plants increased on average by 34 and 55% approximately 24 h after re-watering, while it was 55 and 222% in W–plants, respectively (Table 2). Osmotic potential of hemiparasites of W+ and W–increased on average by 28 and 31% (Table 2), respectively. Interestingly, there was a significant interactive effect of irrigation treatment and water saturation (S4 Table), which might indicate an physiological adjustment to water stress in W–hemiparasites.

### Stable isotopes and stomatal density

**Fig 4:**
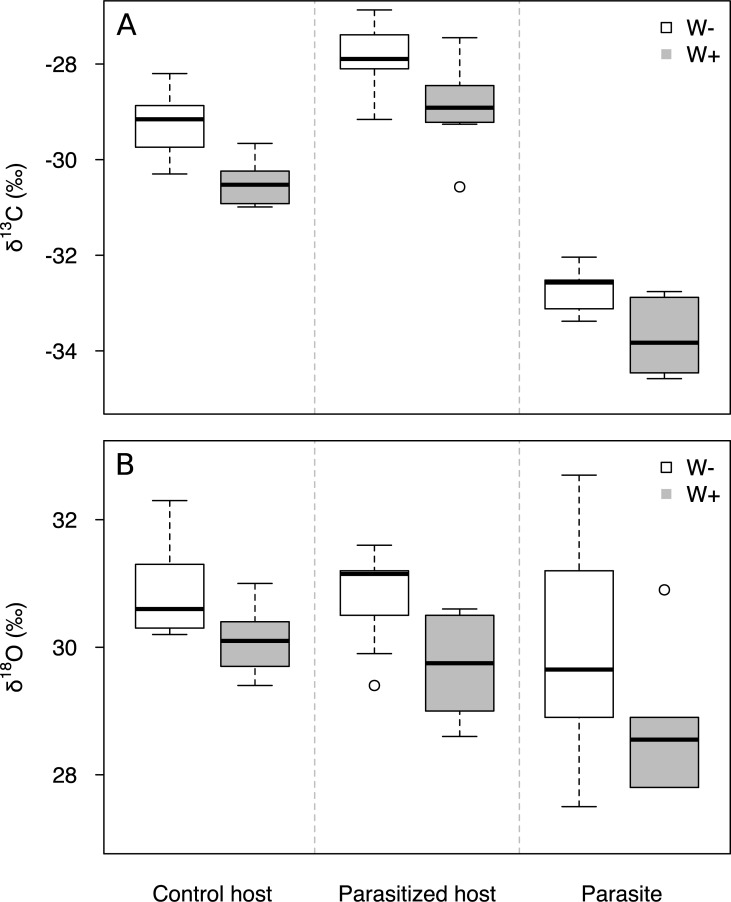
**Carbon (A) and oxygen (B) stable-isotopic composition of above-ground biomass of control and parasitized *Triticum aestivum* host and the hemiparasitic *Rhinanthus alectorolophus* grown under high (W+) and low (W–) irrigation treatments.** n = 10 for both control and parasitized pots and each water treatment.

Biomass of the parasite from W–irrigation treatment was significantly enriched in ^13^C, but only slightly in ^18^O (Table 1; Fig 4) compared with its biomass from W+ treatment. Biomass of the host from W–treatment was significantly enriched in both ^13^C and ^18^O (Table 1; Fig 4). δ^13^C of host biomass was in addition positively affected by parasitism (Table 1). Biomass of the parasite was less enriched in ^13^C and ^18^O than the biomass of parasitized hosts regardless of treatment (t_19_ = –28.39, P<0.001; t_19_ = –2.14, P = 0.046; S5 Table).

Plants grown under contrasting water treatments did not significantly differ in the density of stomata on their leaves and bracts (Figures A and B in S3 Fig; S6 Table).

## Discussion

Both parts of our experimental work focusing on seedlings and adults of hemiparasitic *R*. *alectorolophus* brought novel insights into understanding of the water-stress ecophysiology of root-hemiparasitic plants. For the first time we experimentally showed the sensitivity of *Rhinanthus* seedlings to drought. The seedling survival of first two weeks after their transplant was strongly lowered by drought stress as we hypothesized, in contrast to two-days-older wheat hosts. Our observations also suggest that seedlings might be drought-sensitive both before and shortly after attachment to the host if we assume B-pots already host-connected. Drought stress is likely the mechanism behind field observations documented pronounced mortality of seedlings during spring droughts causing frequent population fluctuations [23,38,39]. Frequency and intensity of such fluctuations may increase in future as a result of climate change, which may eventually cause *Rhinanthus* extinction in some areas.

Similarly to *Rhinanthus* seedlings, water stress negatively affected adult hemiparasites in terms of biomass production and physiological functioning. Water shortage inhibited the hemiparasite’s gas exchange, but drought-stressed and even wilted plants from both treatments still photosynthesized and transpired considerably (Table 2; Fig 3). A negative effect of drought on photosynthetic performance is well recognized for parasitic [40,41] and many non-parasitic plants [42–44] and mostly attributed to reduced stomatal and mesophyll conductance, and to a lesser extent to biochemical limitations [42,45,46]. We could not evaluate the importance of these, as we measured only transpiration rate consisting of stomatal conductance and evaporation of guttation water from hydathode trichomes, which cannot be separated from each other. Interestingly, the ability of wilted hemiparasites to carry out photosynthesis of substantial rate indicates that their guard cells were still turgid and stomata open.

The absence of significant differences in the water-relation parameters between the irrigation treatments (Table 2) indicated that low-irrigated plants did not osmotically adjust to long-term water stress which contrasts with our original hypothesis. We expected these plants to lower their osmotic and water potential as frequently observed in non-parasitic plants responding to drought stress [47–49]. The absence of any osmotic adjustment may refer to limited capacity of *Rhinanthus* to acclimatize to water stress or more probably its capability to tolerate drought without a need to adjust. The latter is supported by the fact that *Rhinanthus* of either treatment had rather low osmotic potential at full turgor (ψπ FT = –1.38 MPa in average; Table 2). Compared to non-parasitic species of similarly dry habitats, e.g. semiarid grassland dicots [50], this value may be low enough to ensure good physiological adjustment to water stress.

The photosynthetic rate of the flowering hemiparasites was affected by the irrigation treatment despite the absence of corresponding osmotic adjustment. The photosynthetic rate in low-irrigated plants (Table 2; Fig 3) was elevated compared with W+ plants of the same osmotic potential and these plants could therefore obtain more autotrophic carbon. This result is unexpected and can be related to a differential acclimation of water-stressed plants to repeated cycles of water stress sometimes referred to as stress memory [51,52]. Studies comparing plants acclimated and not-acclimated to drought stress are rare. Recently, Menezes-Silva et al. [53] reported elevated photosynthesis in coffee plants that underwent multiple drought events and attributed it to biochemical adjustments (e.g. increased activity of Rubisco). Similar evidence had earlier been suggested to be associated with the maintenance of higher electron transport rates in plants acclimated to drought stress [49]. Increased photosynthetic rate may also be related to increased chlorophyll [54] and/or Rubisco concentrations in the hemiparasite underpinned by enhanced resource uptake from host root system under drought conditions [6]. This may be caused by delayed stomatal closure in the hemiparasites grown under W–relative to W+ hemiparasites of the same water status and relative to the host when the hemiparasite acts as a strong sink. Alternatively, allocating more assimilates into roots, the host might facilitate its resource uptake by providing more space for the establishment of haustorial connections and thus enables the hemiparasite to acquire nutrients essential for building up chlorophyll and/or Rubisco molecules.

Lower enrichment of the hemiparasite biomass in ^13^C and ^18^O compared with that of the host corresponds to the water-wasting physiological strategy of rhinanthoid root hemiparasites associated with higher transpiration and lower water-use efficiency (WUE). A similar pattern was reported in a field study from arid areas of Western Australia by Cernusak et al. [55], but the isotope proportions seem to highly depend on particular growing conditions. This might be a reason why non-significant differences in ^13^C were found between *R*. *alectorolophus/Euphrasia rostkoviana* and wheat [5]. The lack of differences in ^13^C in root-hemiparasite–C_3_ host pair was also reported for *Striga-gesnerioides–Vigna-unguiculata* [56] *a*nd *Olax-phylanthi–*multiple-hosts [57]. Similar enrichment in *Olax* and their hosts was explained by their similar WUE [57]. The evaluation of δ^13^C results is further complicated by heterotrophic C uptake by *Rhinanthus*, which might underestimate the differences in WUE between species as suggested by Cernusak et al. [55].

Although the physiological functioning of hemiparasitic and host plants differ in many aspects, both species seem to respond to water stress conditions in a similar way, contrasting to what was previously suggested [6,13]. Hemiparasites and hosts close stomata under severe water stress, increasing their WUE and restricting transpiration and stomatal conductance, which is inferred from higher enrichment of both species biomass in ^13^C and ^18^O under W–(Fig 4). While stomatal closure is ABA-mediated in the host, it is likely that stomata of the hemiparasite close passively after turgor of guard cells is lost. Interestingly, gas exchange of wilted hemiparasites recorded here (Fig 3) demonstrate that the passive stomatal closure is preceded by leaf turgor loss in *R*. *alectorolophus*. Passive stomatal closure was reported to prevail active ABA-mediated stomatal closure in ferns and lycophytes [58], and also in woody angiosperms [59,60], but these two processes might operate in the same species together [61]. This is unlikely to happen in *Rhinanthus* since their stomata actively close only in response to extremely high concentration of ABA [12]. Despite the absence of ABA-mediated stomatal regulation in attached *Rhinanthus*, these plants are known to contain unusually high ABA concentration [24]. ABA may thus contribute to the acclimation of the hemiparasites to drought stress via the formation of dehydrins or other drought-protective proteins as suggested by Jiang et al. [14], but its exact mechanism remains unknown.

In summary, we demonstrated that the adult hemiparasites have certain capacity to withstand drought stress. Their physiological traits, in particular generally low osmotic potential associated with the ability to recover from the wilting, are likely crucial for their growth in moderately dry habitats [22,62]. However, most root-hemiparasitic species of temperate grasslands (including those of the genus *Rhinanthus*) display rather prominent limit of their ecological niche at the dry-end of the water availability gradient. The lack of further physiological adjustment to more severe drought demonstrated here may thus cause a substantial decline of the hemiparasitic species under the projected (and recently also observed) climate change-induced increase of temperature and drought events [63]. Nevertheless, more studies on water-relations of root hemiparasites under repeated drought stress are needed to accurately estimate the stability of their populations in future warmer climate.

## Supporting information

S1 Fig **Höfler plot (A) and pressure–volume curve (p–v curve, B) revealing water relation parameters of flowering *Rhinanthus alectolophus*.** The turgor loss point, which is usually defined as the first point of linearly decreasing segment of a p–v curve (light grey), was hard to determine due to hyperbolic shape of the p–v curve. To reduce the error of the determination, we replaced it by a corrected turgor loss point (dark grey) corresponding to 10% of full turgor (Ψ_p_). Additionally, the intercept of the linear segment of the p–v curve with the x-axis was fixed to 23.1% of total RWC (RWC_AW_) estimated to represent the volume of apoplastic water inside measured plant (B). This enabled us to define the linear segment, i.e. osmotic potential and hence other parameters more precisely. Ψ = water potential, Ψ_p_ = turgor or pressure potential, Ψ_π_ = osmotic potential, Ψ_π FT_ = osmotic potential at full turgor (black triangle), Ψπ_TLP_ = osmotic potential at turgor loss point, RWC = relative water content, RWC_AW_ = fraction of apoplastic water fixed at 23.1% of total RWC, RWC_TLP_ = RWC at turgor loss point, RWC_TLPcor_ = RWC at corrected turgor loss point, ε = the modulus of elasticity.
(PDF)

S2 Fig **The recovery of wilted *Rhinanthus alectorolophus* (A and C) from severe drought stress several hours after re-watering (B and D).** One non-flowering and one flowering individuals are shown. Note the effect of drought stress on wheat, which was used as a host species.
(PDF)

S3 Fig **Stomatal density on leaves (A) and bracts (B) of hemiparasitic *Rhinanthus alectorolophus* grown under high (W+) and low irrigation treatments (W–).** Both adaxial and abaxial sides are presented. n = 9 for W+ leaves, n = 5 for W–leaves, n = 9 for W+ bracts, and n = 7 for W–bracts.
(PDF)

S1 Table ANOVA table of linear models describing the effects of irrigation treatment, infection by the parasitic *Rhinanthus alectorolophus*, harvest day, and their interactions on the host (*Triticum aestivum*) above-ground biomass and its stable-isotopic composition. Factor Infected represents the effect of parasitic infection on host parameters. δ13C and δ18O represent the isotopic composition of host overall biomass. The effects not tested for a particular variable are indicated by light grey. Significant terms (P<0.05) are in bold. df: degrees of freedom; SS: sum of squares; F: F-statistics; p: significance level.
(PDF)

S2 Table ANOVA table of linear models describing the effect of irrigation treatment on the above-ground biomass and its stable-isotopic composition of parasitic *Rhinanthus alectorolophus*. δ^13^C and δ^18^O represent the isotopic composition of the parasite biomass. Significant terms (P<0.05) are in bold. df: degrees of freedom; SS: sum of squares; F: F statistics; p: significance level.
(PDF)

S3 Table ANOVA table of linear models describing the effects of irrigation treatment, osmotic potential of leaves subjected to gas exchange measurements, and their interaction on photosynthetic and transpiration rate of parasitic *Rhinanthus alectorolophus*. Significant terms (P<0.05) are in bold. df: degrees of freedom; F: F statistics; p: significance level.
(PDF)

S4 Table ANOVA table of linear models describing the effects of irrigation treatment, leaf water saturation, and their interaction on photosynthetic and transpiration rate of parasitic *Rhinanthus alectorolophus*. Factor Saturated represents the effect of leaf saturation by water on the hemiparasite parameters. Significant terms (P<0.05) are in bold. df: degrees of freedom; F: F statistics; p: significance level.
(PDF)

S5 Table ANOVA table of linear models describing the effects of irrigation treatment, plant species, and their interaction on the stable-isotopic composition (δ^13^C and δ^18^O) of host (*Triticum aestivum*) and parasite (*Rhinanthus alectorolophus)* above-ground biomass. Factor Plant represents the effect of plant species on isotopic parameters. δ^13^C and δ^18^O represent the isotopic composition of plant biomass. Significant terms (P<0.05) are in bold. df: degrees of freedom; F: F statistics; p: significance level.
(PDF)

S6 Table ANOVA table of linear models describing the effects of irrigation treatment and sampling place (leaf/bract) on the density of stomata on abaxial and adaxial leaf and bract sides of parasitic *Rhinanthus alectorolophus*. Factor Leaf/bract represents the effect of sampling place on stomatal density. Significant terms (P<0.05) are in bold. df: degrees of freedom; F: F statistics; p: significance level.
(PDF)

S1 File Seedling survival data and all data collected from adult plants, except for pressure-chamber data. D and W refer to parasitized low and high irrigation pots. HD and HW refer to non-parasitized low and high irrigation pots. Additional abbreviations used are explained in individual data sheets (in green).
(XLS)

S2 File Pressure-chamber data. Each plant measurement is displayed on a separate sheet. D and W refer to parasitized low and high irrigation pots. D21 measurement was used for Figures A and B in S1 Fig.
(XLSX)
