# Supplementary figures and images for: Water-stress physiology of Rhinanthus alectorolophus, a root-hemiparasitic plant
Source: PLoS One. 2018 Aug 1;13(8):e0200927. doi: 10.1371/journal.pone.0200927 (PMC6070206; doi:10.1371/journal.pone.0200927)

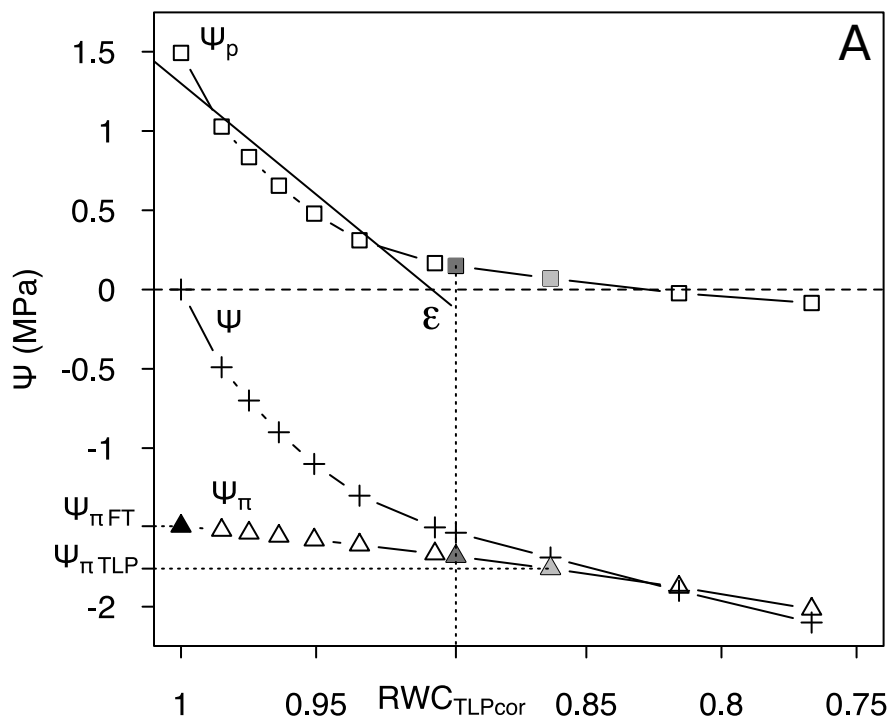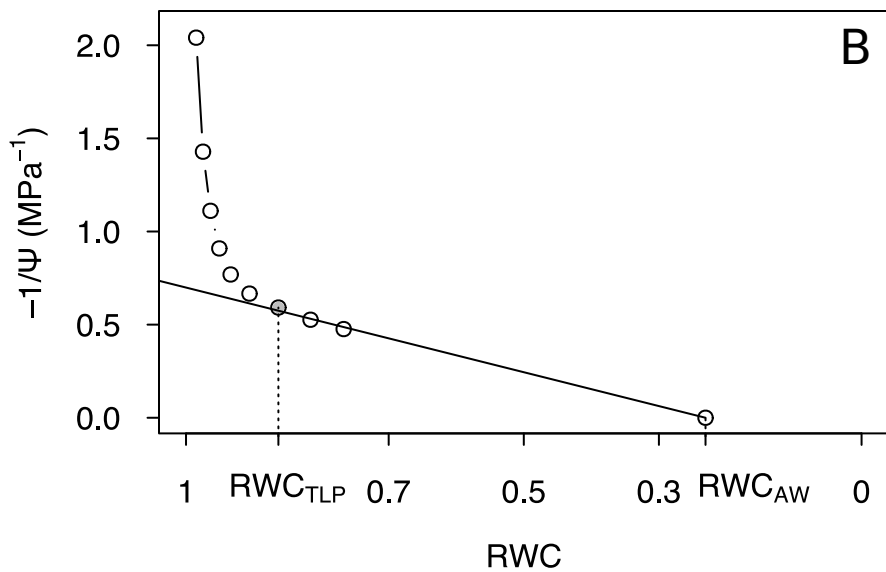

Supplement: S1 Fig — Höfler plot (A) and pressure–volume curve (p–v curve, B) revealing water relation parameters of flowering Rhinanthus alectolophus. The turgor loss point, which is usually defined as the first point of linearly decreasing segment of a p–v curve (light grey), was hard to determine due to hyperbolic shape of the p–v curve. To reduce the error of the determination, we replaced it by a corrected turgor loss point (dark grey) corresponding to 10% of full turgor (Ψp). Additionally, the intercept of the linear segment of the p–v curve with the x-axis was fixed to 23.1% of total RWC (RWCAW) estimated to represent the volume of apoplastic water inside measured plant (B). This enabled us to define the linear segment, i.e. osmotic potential and hence other parameters more precisely. Ψ = water potential, Ψp = turgor or pressure potential, Ψπ = osmotic potential, Ψπ FT = osmotic potential at full turgor (black triangle), ΨπTLP = osmotic potential at turgor loss point, RWC = relative water content, RWCAW = fraction of apoplastic water fixed at 23.1% of total RWC, RWCTLP = RWC at turgor loss point, RWCTLPcor = RWC at corrected turgor loss point, ε = the modulus of elasticity. (PDF) [file pone.0200927.s001.pdf]

A

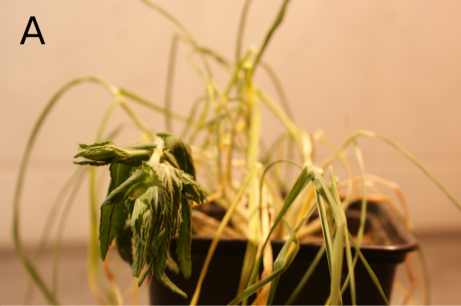

C

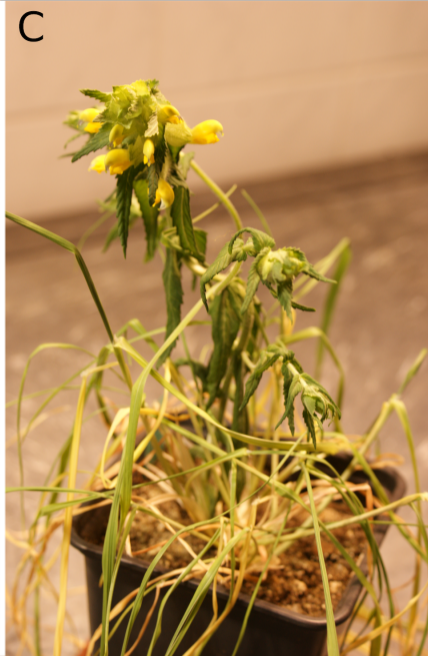

D

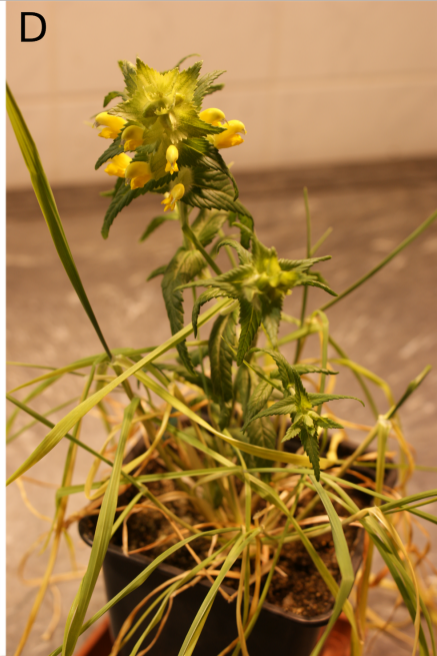

B

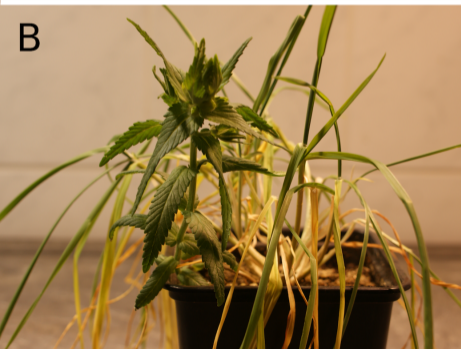

Supplement: S2 Fig — The recovery of wilted Rhinanthus alectorolophus (A and C) from severe drought stress several hours after re-watering (B and D). One non-flowering and one flowering individuals are shown. Note the effect of drought stress on wheat, which was used as a host species. (PDF) [file pone.0200927.s002.pdf]

Stomatal density per mm<sup>2</sup>

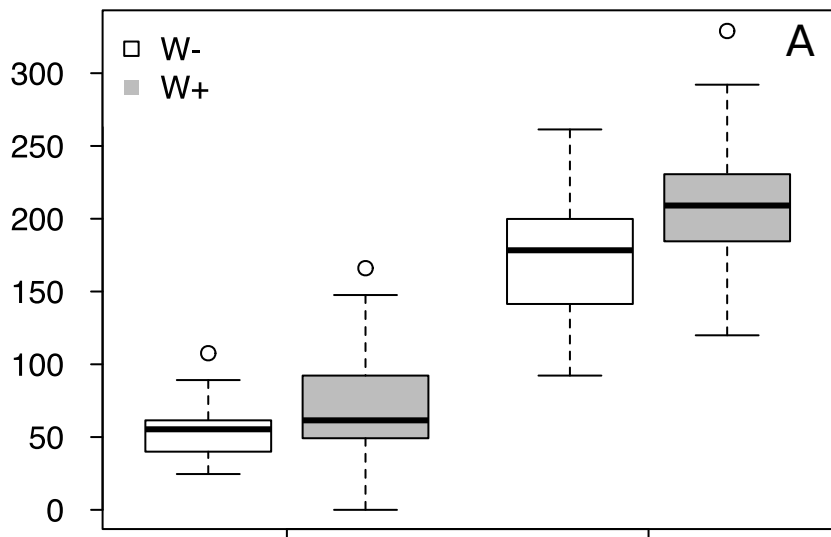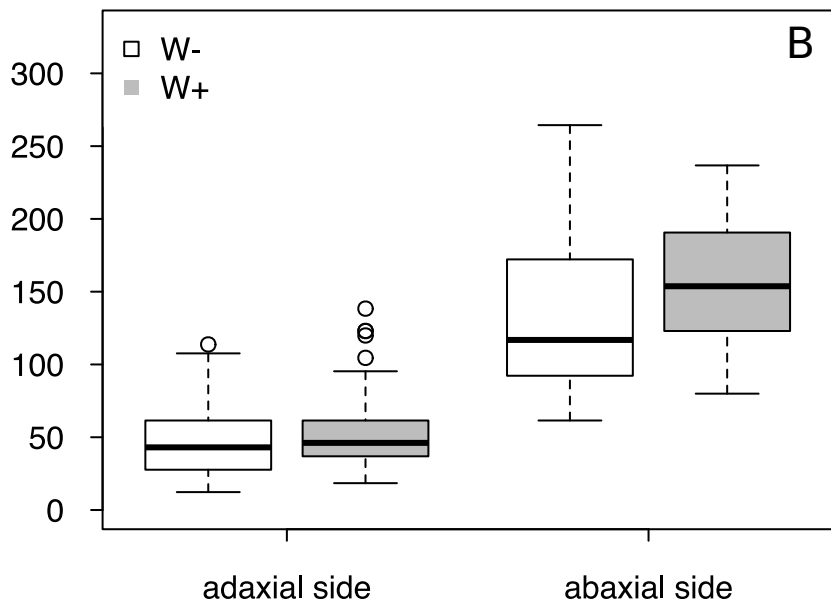

Supplement: S3 Fig — Stomatal density on leaves (A) and bracts (B) of hemiparasitic Rhinanthus alectorolophus grown under high (W+) and low irrigation treatments (W–). Both adaxial and abaxial sides are presented. n = 9 for W+ leaves, n = 5 for W–leaves, n = 9 for W+ bracts, and n = 7 for W–bracts. (PDF) [file pone.0200927.s003.pdf]
