# Supplementary material for: Water-stress physiology of Rhinanthus alectorolophus, a root-hemiparasitic plant
Source: PLoS One. 2018 Aug 1;13(8):e0200927. doi: 10.1371/journal.pone.0200927 (PMC6070206; doi:10.1371/journal.pone.0200927)
Supplement: S1 Table — Factor Infected represents the effect of parasitic infection on host parameters. δ13C and δ18O represent the isotopic composition of host overall biomass. The effects not tested for a particular variable are indicated by light grey. Significant terms (P<0.05) are in bold. df: degrees of freedom; SS: sum of squares; F: F-statistics; p: significance level. (PDF) [file pone.0200927.s004.pdf]

S1 Tab

	Host biomass (parasitized and control)				Host control biomass				Host δ^{13}C				Host δ^{18}O			
Effect	*df*	*SS*	*F*	*P*	*df*	*SS*	*F*	*P*	*df*	*SS*	*F*	*P*	*df*	*SS*	*F*	*P*
Treatment	**1,58**	**2.44**	**9.24**	**0.004**	**1,18**	**1.02**	**29.82**	**3.46 x 10^{-5}**	**1,36**	**11.69**	**24.90**	**1.55 x 10^{-5}**	**1,36**	**7.83**	**18.01**	**1.47 x 10^{-4}**
Infected	**1,58**	**20.94**	**79.34**	**1.9 x 10^{-12}**					**1,36**	**21.37**	**45.55**	**7.02 x 10^{-8}**	1,36	0.31	0.70	0.41
Harvest day	1,58	0.02	0.06	0.81												
Treatment × Infected	1,58	0.01	0.02	0.88					1,36	0.04	0.78	0.38	1,36	0.34	0.79	0.38
Treatment × Harvest day	1,58	0.01	0.03	0.86												
