# Supplementary material for: Water-stress physiology of Rhinanthus alectorolophus, a root-hemiparasitic plant
Source: PLoS One. 2018 Aug 1;13(8):e0200927. doi: 10.1371/journal.pone.0200927 (PMC6070206; doi:10.1371/journal.pone.0200927)
Supplement: S2 Table — δ13C and δ18O represent the isotopic composition of the parasite biomass. Significant terms (P<0.05) are in bold. df: degrees of freedom; SS: sum of squares; F: F statistics; p: significance level. (PDF) [file pone.0200927.s005.pdf]

S2 Tab

	Parasite biomass				Parasite $\delta^{13}C$				Parasite $\delta^{18}O$			
Effect	*df*	*SS*	*F*	*P*	*df*	*SS*	*F*	*P*	*df*	*SS*	*F*	*P*
Treatment	**1,42**	**0.97**	**13.17**	**7.6×10^{-4}**	**1,18**	**4.78**	**13.31**	**0.002**	1,18	8.06	4.38	0.051
