# Supplementary material for: Water-stress physiology of Rhinanthus alectorolophus, a root-hemiparasitic plant
Source: PLoS One. 2018 Aug 1;13(8):e0200927. doi: 10.1371/journal.pone.0200927 (PMC6070206; doi:10.1371/journal.pone.0200927)
Supplement: S3 Table — Significant terms (P<0.05) are in bold. df: degrees of freedom; F: F statistics; p: significance level. (PDF) [file pone.0200927.s006.pdf]

S3 Tab

	Photosynthesis			Transpiration		
Effect	*df*	*F*	*P*	*df*	*F*	*P*
Treatment	**1,21**	**13.47**	**0.001**	1,21	0.42	0.52
Osmotic potential ($\Psi_{\Pi \text{ gas exchange}}$)	**1,21**	**51.49**	**<0.001**	**1,21**	**40.22**	**<0.001**
Treatment × Osmotic potential ($\Psi_{\Pi \text{ gas exchange}}$)	1,21	1.31	0.27	1,21	0.15	0.70
