# Supplementary material for: Water-stress physiology of Rhinanthus alectorolophus, a root-hemiparasitic plant
Source: PLoS One. 2018 Aug 1;13(8):e0200927. doi: 10.1371/journal.pone.0200927 (PMC6070206; doi:10.1371/journal.pone.0200927)
Supplement: S4 Table — Factor Saturated represents the effect of leaf saturation by water on the hemiparasite parameters. Significant terms (P<0.05) are in bold. df: degrees of freedom; F: F statistics; p: significance level. (PDF) [file pone.0200927.s007.pdf]

S4 Tab

	Photosynthesis			Transpiration		
Effect	*df*	*F*	*P*	*df*	*F*	*P*
Treatment	**1,21**	**7.07**	**0.015**	1,21	0.32	0.58
Saturated	**1,21**	**35.06**	**<0.0001**	**1,21**	**57.27**	**<0.0001**
Treatment × Saturated	1,21	3.59	0.07	**1,21**	**8.84**	**0.007**
