# Supplementary material for: Water-stress physiology of Rhinanthus alectorolophus, a root-hemiparasitic plant
Source: PLoS One. 2018 Aug 1;13(8):e0200927. doi: 10.1371/journal.pone.0200927 (PMC6070206; doi:10.1371/journal.pone.0200927)
Supplement: S5 Table — Factor Plant represents the effect of plant species on isotopic parameters. δ13C and δ18O represent the isotopic composition of plant biomass. Significant terms (P<0.05) are in bold. df: degrees of freedom; F: F statistics; p: significance level. (PDF) [file pone.0200927.s008.pdf]

S5 Tab

	$\delta^{13}C$			$\delta^{18}O$		
Effect	*df*	*F*	*P*	*df*	*F*	*P*
Treatment	**1,18**	**766.12**	**<0.0001**	**1,18**	**4.76**	**0.043**
Plant	**1,18**	**12.73**	**0.002**	**1,18**	**8.87**	**0.008**
Treatment × Plant	1,18	0.07	0.80	1,18	1.80	0.20
