# Supplementary material for: Water-stress physiology of Rhinanthus alectorolophus, a root-hemiparasitic plant
Source: PLoS One. 2018 Aug 1;13(8):e0200927. doi: 10.1371/journal.pone.0200927 (PMC6070206; doi:10.1371/journal.pone.0200927)
Supplement: S6 Table — Factor Leaf/bract represents the effect of sampling place on stomatal density. Significant terms (P<0.05) are in bold. df: degrees of freedom; F: F statistics; p: significance level. (PDF) [file pone.0200927.s009.pdf]

S6 Tab

	Stomatal density (adaxial)			Stomatal density (abaxial)		
Effect	*df*	*F*	*P*	*df*	*F*	*P*
Treatment	1,16	0.21	0.65	1,15	3.25	0.09
Leaf/bract	**1,131**	**23.49**	**<0.0001**	**1,116**	**53.85**	**<0.0001**
